# Opposing the toxic apartheid: The painted veil of the COVID‐19 pandemic, race and racism

**DOI:** 10.1111/gwao.12523

**Published:** 2020-09-06

**Authors:** Grace Gao, Linna Sai

**Affiliations:** ^1^ Newcastle Business School Northumbria University UK; ^2^ Huddersfield Business School University of Huddersfield UK

**Keywords:** Chinese, coronavirus, identity, racism, vulnerability

## Abstract

This article is a personal reflection of how the coronavirus exposes ‘shocking’ levels of racism against us, and our vulnerability as Chinese women living in Britain. By reflecting our experiences of verbal and physical race‐based violence connected to coronavirus, we explore the fluidity of our racial identities, the taken‐for‐granted racial stereotypes and white privilege, and everyday racism in the UK. Can the vulnerable use vulnerability as an agent to shift the moment of helplessness? We contribute to the uncomfortable yet important debate on racism against Chinese women living in the UK through voicing up our embodied vulnerability as invisible and disempowered subjects to this viral anti‐Chinese racism. This is a form of resistance where we care for the racialized and marginalized others. In doing so, we lift the painted veil of the pandemic, race and racism to collectively combat racial inequalities.

## 1 WE ARE NOT VIRUSES!

We are ‘sitting ducks’ during this global health crisis. An increased tide of race‐based violence and animosity toward the Chinese are flashing up, in the aftermath of the coronavirus outbreak. We were told to go home. It tortures us. Whilst we are hesitant to risk our bodies being assaulted physically and verbally, it takes courage to unveil our vulnerabilities as the ‘targets’ of this viral racist attack associated with ‘Chinese virus’ and ‘Kung flu’ — racist terms publicly promoted by politicians such as US President Trump (*Guardian*, 2020a). The term ‘Kung Fu’ represents a time‐honoured tradition grounded in fortitude and courage, extracted from Chinese traditional martial arts (Yam, 2020). Over time, it has been frequently used as an expression in honour of any discipline or skill that is achieved by constant hard work and practice. But now it has become a racist joke. The reason for us being attacked is simply because of our race and skin tone. It is appalling for us to be seen as unhygienic, filthy and unsanitary, and being accused of carriers of the deadly virus; some people even connected us to the ‘Yellow Peril’ theme which portrays us as an image of ‘evil’ and ‘aliens’. By reflecting the ample manifestations of maskaphobia, prejudice and racial discrimination connected to the coronavirus against Chinese in Britain, we break our silence and speak up against the increasing anti‐Chinese rhetoric, with whom we are in solidarity and to whom we offer our labour, time and support as a driver of resistance.

## 2 VIOLENCE AND VULNERABILITY

Since the outbreak of the novel coronavirus, anti‐Chinese sentiment has been spreading around the world. In the UK, incidents of xenophobia and racist harassment against Chinese have significantly increased. A recent poll into racism suggests that 76 per cent ethnic Chinese have experienced a direct racial slur (at least once), making us the most common victims of racism in the UK (CGTN, 2020). A Chinese postgraduate student was reported being verbally and physically abused for wearing a face mask when she was walking alone in Sheffield (Jones, 2020). In Leicestershire, another two Asian students who were mistakenly thought to be Chinese, were pelted with eggs on the street (*Guardian*, 2020b). In the schools, racists have also targeted Chinese kids by expressing slogans such as ‘keep away, they are all poisoned’ that defame our community. However, this is not the end.

*Since the initial case was confirmed in the UK on January 31* ^*st*^ *, 2020, the city where I live has been fuelled with a surging increase of race‐based violence. I have been warned by other fellow Chinese in the local community who have been spat at on the street, been shouted at as a liar. Soon I become very cautious when walking out. I avoided the narrow alleys even during the daytime and I kept my head down, lay low and walked fast.*

Such risks of being physically abused are interwoven with the mainstream media and we are becoming the target for cyber racist violence. A Danish newspaper, *Jyllands‐Posten*, published a satirical cartoon of the Chinese national flag, replacing the five gold stars by the deadly virus, which implies that the Chinese should be blamed for this outbreak — an insult to China (BBC, 2020). In the past, Chinese groups have been depicted as intelligent, hardworking and compliant as part of a diaspora (e.g., Archer, Francis, & Mau, 2010; Costigan, Hua, & Su, 2010). A discourse of ‘model minority’ was also observed in Britain (Yeh, 2014). However, under the current pandemic, the public perception of Chinese as ‘model minority’ has been shifted to the ‘yellow perils’ (Lee, 2020).

*I was shocked by the idiocy of anti‐China sentiment and racist memes based on ignorance such as ‘Chinese eat bats’ ‘Chinese eat dogs’. Chinese is seen as the source and carrier of this deadly virus. I felt very disgusted. I soon learned that the aggressive comments I received on my social media account comes from a ‘bat soup’ video that purported bat as a Chinese dish. And I was even being asked if that is true by a person who has known me for at least two years, asking if I do eat these things. I responded that Chinese only eat what everyone else eats and muted the person since then.*

Ironically, only certain epidemic virus outbreaks, such as SARS which originated from China and Ebola from Africa, have led to a racial backlash. However, neither the swine flu (H1N1) epidemic which originated from the United States nor the mad cow disease from Britain has been racialized in the past. Prejudice and ethnocentrism toward us, deliberately dehumanize Chinese people and imply that it is our fault. Such prejudice also carries to our offline interactions and behaviours.

*Before the national lockdown, I had dinner in London's Chinatown. It was unusually quiet. I entered one restaurant which is apparently less busy than usual. I had a chat with the manager, and believed that Western people were misled by the false news of ‘Chinese eat bats’ and scared of eating Chinese food.*

However, there is no evidence that eating Chinese food will cause people to get infected, which is supported by the World Health Organization's (WHO) claim that people will be likely to get infected by close contact/interaction with people who have been exposed to the virus.

Our captured moments continue to unravel the ample manifestations of maskaphobia associated with behavioural changes in response to the Yellow attacks.

*In early February, few weeks before the national lockdown, I had been shoved and shouted at, calling me ‘virus’ while walking on the street. I become more cautious afterwards by walking faster and avoiding contact with other people. At the time, I did not wear a mask because I was afraid of being physically assaulted over maskaphobia. But I was also afraid of being infected through droplets.*

*When I started to wear a mask, it felt like I become a target of public criticism, even though it was common to wear a mask in Italy — the Europe epicentre. I have learnt from other countries where the local authorities have advised citizens to wear face masks to protect ourselves and others. But the unwanted attention made me feel uncomfortable. I then stopped wearing a mask until the government's announcement on 4th June 2020 that it is compulsory to wear face coverings on public transports and advised to wear face coverings at places where social distancing is difficult to maintain. After that I feel safe to wear a mask.*

How can we mobilize our vulnerability (Butler, 2016)? Gender theorist Judith Butler's keynote speech at the international conference of ‘How to Act Together: From Collective Engagement to Protest’ in Belgrade, Serbia, reminded us to reconsider and rethink the relationship between vulnerability and resistance that:*we may think that either we are vulnerable or we resist, and that resistance consists of overcoming vulnerability, [but] the short version of my argument is that resistance can be a way of mobilising vulnerability, and that even fighting back or triumphing in a fight does not negate vulnerability.*What is at stake under the current pandemic, is that we, as human beings, seek to become secure, yet our embodied vulnerabilities are emerging. Vulnerability as resistance matters (Ahonen et al., 2020; Gilmore, Harding, Helin, & Pullen, 2019; Helin, 2019), because it has become our only choice of seeking to foreground and oppose the increased condition of precariousness.

In resonating with Simone de Beauvoir's feelings of ‘irritation’ and ‘hesitation’ before writing *The Second Sex*, where she described her long‐time hesitation of writing a book on women — a subject that is irritating especially to women and is not new — we find ourselves choked with emotions to write this piece. We are provoked by Kyoo Lee's (2013) self‐question and reflection upon herself ‘feeling being a problem [as an Asian female], [here], not feeling or being a problem but “feeling being” one’ (p. 87). Women of Asian origins have been depicted as exotic, submissive sex objects and invisible (e.g., Mukkamala & Suyemoto, 2018). These stereotypes constructed by the dominant society group ‘legitimise’ us as controllable and deferential — we are expected to be ‘OK with anything’, be quiet and not speak up for ourselves. The reflection of feeling being a problem originating from embodied vulnerability is further exaggerated when framed, targeted and staged by unexpected and uncontrollable forces in the workplace.

*Recently, the vice‐chancellor of my university announced a voluntary redundancy package in response to the financial hardship as an effect of the pandemic. Several emails were followed up by the Dean of the school and the head of department, encouraging staff to discuss the package with their line managers or head of departments. I couldn’t help worrying about my job and wondering whether there would be compulsory redundancies if no one took the package. Obviously, it is a difficult time for the higher education sector and there are limited job openings. This will result in too many academics competing for one position. As a junior female academic, I would not be able to compete with other white British counterparts.*

Concerns about job security in white institutions have exacerbated our irritation in challenging the normalized practices in the neoliberal academia. However, due to precarious positioning, we are forced to navigate ourselves strategically with the whiteness to survive.

## 3 RACE AND EVERYDAY RACISM

People from Chinese backgrounds have been constructed as ‘different’, ‘aliens’ and ‘foreigners’ by the hegemonic perpetuation of dominant ethnic (white) groups (Cheng, 2013; Liu, 2017a, 2017b). Despite us being assimilated into British society, we are still ‘outsiders’ and being excluded from the mainstream narratives depicted by the dominant societal groups.

*In response to the increased racist attacks, I received a message circulated from the top level, urging staff to support their Chinese colleagues and students. Yet one of our students was involved in posting offensive materials that may constitute a hate crime. In the footage, the student said, ‘I am rich, white, privileged, and tanned … and got good teeth.’ This was reported immediately to the police and is now the subject of a police investigation.*

White power constantly ‘passes’ through our bodies as people of colour, who have been instrumentalized into everyday white supremacist practices (Bonilla‐Silva, 2012). A burgeoning racial discrimination and injustice are manifested under the white supremacist ideology (e.g., Burnett, 2017). The entrenched system of government authorities constantly reinforces the racialized climate injustice and a bespoke antagonistic ‘global policing’ for immigration control at the expense of the ‘others’ (Bowling & Westenra, 2018; Sealey‐Huggins, 2018).

*Ironically, as Chinese, I have not been considered as a ‘minority’ from the eyes of other groups. I always hear ‘Oh, China grows fast and is rich.’ I remembered my recent conversation with a Black colleague about Chinese ability in building an insta‐hospital in 10 days to battle the coronavirus. And he didn’t think people who come from such a nation with great strength and wealth could have faced social inequality and inequity like they have faced. Likewise, white British also don’t believe that Chinese would experience racism or simply deny it.*

We are stuck in the middle of White and Black, West and East — always as the ‘betweeners’. The visibility of Chinatowns, the increasing numbers of Chinese students studying across British institutions, and Chinese workers' higher average earnings compared to their white British counterparts (Office for National Statistics, 2019) inevitablycontribute to the ethnic Chinese being omitted from the discourse about race, racism and inequality. Yet Chinese are at the bottom of ‘displays of power’ manifested in both social and business settings, where a society is perpetuated by white supremacist ideologies (Dar, Liu, Dy, & Brewis, 2020).

*Following the shocking news of the killing of George Floyd, an initiative in establishing BAME staff network was circulated in my school. These ‘anti‐racist’ messages were sent by white people from the top level to promote equality and equity policies for the sake of charters. For example, promoting Athena SWAN related projects for materialist ranking purposes. But I know nothing will actually change, so I keep silent.*

Such paradoxical feelings present a continued hesitation to stand up against how the dominant white constructs whiteness as moral authority to ‘correct’ the underrepresented minorities. White supremacy is frequently cited to describe the centuries‐old racialized social system (Feagin, 2013) that consists ‘totality of the social relations and practices that reinforce white privilege’ (Bonilla‐Silva, 2006, p. 9). It manifests in the compliance of reproducing and reinforcing white solidarity and white social capitalism and thereby excluding women and people of colour. As such, management and organizational scholars call for rethinking racial capitalism and redefining differences by claiming that capitalism is racist and advocating for acting up for resistance (e.g., Bhattacharyya, 2018; Dar et al., 2020; Lorde, 2017).

Bronwyn Frederick (2010) also writes on the different ways that a marginalized other can resist white dominance, saying that: *there are those who are undertaking [resistance] through the legal and political rights arenas both nationally and internationally, those who write about it through analytically researched academic papers challenging the status quo, and others who do so through creative narrative prose and pieces of art.* (p. 548) It becomes clear that there are no right or wrong ways of doing resistance as long as we are resisting. We stand together, write together and resist together.

## 4 EXPRESSING THE UNEXPRESSED

It is a struggle to express the inexpressible ‘true’ stories and feelings when writing this article. Chinese, and other East Asians as a whole, have long been criticized in their proximity to white privilege and thereby benefiting from white supremacy (Young, 2015). This is a big lie. As female academics of Chinese origin, choices have to be made between ‘authenticity’ and ‘appropriateness’ to fit in the dominant white scholarly community, due to fear of being harshly criticized.

*Few weeks ago, when I finally completed the marking and moderation process, I received a message from a white senior colleague in a night, implying to raise marks for a student from her class. She explained that the student is a non‐native English speaker from Europe, and markers should be more ‘sympathetic’. I understand her intention, but I believe that I should not lower down the academic bargain simply based on whether English is the student's native language or not. I have provided enough justification to make sense of my scoring, but eventually ‘surrendered’, to stay in the loop and avoid being seen as a ‘troublemaker’.*

The paradox pushes us to relate to others for the benefits of being included in a group. There is a need to perform different facets to different audiences, but at times, we are distanced from the ‘authentic self’. The contradictory effect on (re)constructing our identities can be further reflected through the fluid racial identity process (Helms, 1995).

*Since the pandemic, I started to rethink the idea of racism in the context of my experience. I realised that I have been constantly conforming to the white culture while trivialising the significance of race and racism. I was ‘colour‐blinded’ because I have been trained to learn and express how to speak and think in the ‘language’ of whiteness. The sudden realisation of some racist practices resulting from the pandemic marked my dissonance with an ongoing suspicious mind and anxiety.*

The instabilities of racial identity are presented in the moments of transitioning, where ‘the collective and individual, the past and present, and the central and marginalised identities are spheres and overlap’ (Shirazi, 2018, p. 16). Our changes embedded in these short narratives are juxtaposed within a new cultural, political and economic environment.

Looking back, the recognized race‐based oppressions have been constantly (re)structuring our lives and livelihoods as Chinese women living in the UK. Those neglected attributes and vulnerabilities in the past have been amplified amid this global health crisis. Our account is not just a personal diary. It ‘pushes’ us to rethink and reflect sensitive subjects — race and racism, which might be uncomfortable for the dominance yet have constantly affected our choices for practising silence and/or taking the initiative to voice up as members of ethnic minorities. By writing this piece collectively, it extends beyond the sum of our embodied oppressions and voices seeking to alleviate problematic racism issues, which grounded in British history, for a better and equitable society.

It is time to be bold and brave. Having rolled with the punches for a long time to survive in the neoliberal capitalist society, this article contributes to the burgeoning discussion on race, racism and writing differently as a form of resistance. Writing through vulnerability liberates us to heal, to calm down and to find meanings in our lived experiences. Moving beyond the movement of #BlackLiveMatters, we recognize those disempowered and invisible others alike, in everyday racism; and open up a new dialogue of the toxic racial apartheid amplified during the COVID‐19 pandemic in the UK. After all, the subject of race and racism will never fade away.

## DECLARATION OF CONFLICTING INTEREST

Both authors have nothing to disclose and no conflicts of interest.

## References

[gwao12523-bib-0001] Ahonen, P. , Blomberg, A. , Doerr, K. T. , Einola, K. , Elkina, A. , Gao, G. , … Zhang, L. E. (2020). Writing resistance together. Gender, Work and Organization, 27(4), 447–470. 10.1111/gwao.12441

[gwao12523-bib-0002] Archer, L. , Francis, B. , & Mau, A. (2010). The Culture Project: Diasporic negotiations of ethnicity, identity and culture among teachers, pupils and parents in Chinese language schools. Oxford Review of Education, 36(4), 407–426. 10.1080/03054981003775293

[gwao12523-bib-0003] BBC . (2020). Coronavirus: Denmark in cartoon bust‐up with China over flag. Retrieved from https://www.bbc.co.uk/news/world-europe-51295225

[gwao12523-bib-0004] Bhattacharyya, G. (2018). Rethinking racial capitalism: Questions of reproduction and survival. London, UK: Rowman & Littlefield.

[gwao12523-bib-0005] Bonilla‐Silva, E. (2006). Racism without racists: Colour‐blind racism and the persistence of racial inequality in the United States. Lanham, MD: Rowman & Littlefield.

[gwao12523-bib-0006] Bonilla‐Silva, E. (2012). The invisible weight of whiteness: The racial grammar of everyday life in contemporary America. Ethnic and Racial Studies, 35(2), 173–194.

[gwao12523-bib-0007] Bowling, B. , & Westenra, S. (2018). A really hostile environment: Adiaphorization, global policing and the crimmigration control system. Theoretical Criminology, 24, 163–183.

[gwao12523-bib-0008] Burnett, J. (2017). Racial violence and the Brexit State. Race & Class, 58(4), 85–97. 10.1177/0306396816686283

[gwao12523-bib-0009] Butler, J. (2016). Vulnerability in resistance. Durham, NC: Duke University Press.

[gwao12523-bib-0010] CGTN . (2020). Ethnic Chinese are the most common victims of racism in the UK according to a YouGov poll. Retrieved from https://newseu.cgtn.com/news/2020-06-29/Ethnic-Chinese-most-common-victims-of-racism-in-UK-according-to-poll-RHnkdR3fVu/index.html

[gwao12523-bib-0011] Cheng, W. (2013). Strategic orientalism: Racial capitalism and the problem of ‘Asianness’. African Identities, 11(2), 148–158. 10.1080/14725843.2013.797284

[gwao12523-bib-0012] Costigan, C. L. , Hua, J. M. , & Su, T. F. (2010). Living up to expectations: The strength and challenges experienced by Chinese Canadian students. Canadian Journal of School Psychology, 25(3), 223–245. 10.1177/0829573510368941

[gwao12523-bib-0013] Dar, S. , Liu, H. , Dy, A. M. , & Brewis, D. N. (2020). The business school is racist: Act up! Organization, 1–12. 10.1177/1350508420928521

[gwao12523-bib-0014] Feagin, J. R. (2013). The white racial frame: Centuries of racial framing and counter‐framing. New York, NY: Routledge. 10.4324/9780203076828

[gwao12523-bib-0015] Frederick, B. L. (2010). Empowering ourselves: Australian aboriginal women. Signs, 35(3), 546–550. 10.1086/648511

[gwao12523-bib-0016] Gilmore, S. , Harding, N. , Helin, J. , & Pullen, A. (2019). Writing differently. Management Learning, 50(1), 3–10. 10.1177/1350507618811027

[gwao12523-bib-0017] Guardian . (2020a). Donald Trump calls Covid‐19 ‘kung flu’ at Tulsa rally. Retrieved from https://www.theguardian.com/us-news/2020/jun/20/trump-covid-19-kung-flu-racist-language

[gwao12523-bib-0018] Guardian . (2020b). Chinese in the UK report ‘shocking’ levels of racism after coronavirus outbreak. Retrieved from https://www.theguardian.com/uk-news/2020/feb/09/chinese-in-uk-report-shocking-levels-of-racism-after-coronavirus-outbreak

[gwao12523-bib-0019] Helin, J. (2019). Dream writing: Writing through vulnerability. Qualitative Inquiry, 25(2), 95–99. 10.1177/1077800418810984

[gwao12523-bib-0020] Helms, J. E. (1995). An update of Helm's White and people of color racial identity models. In J. G. Ponterotto , J. M. Casas , L. A. Suzuki , & C. M. Alexander (Eds.), Handbook of multicultural counselling (pp. 181–198). Los Angeles, CA: Sage.

[gwao12523-bib-0021] Jones, S. (2020). Chinese student attacked in Sheffield over coronavirus. *The Star.* Retrieved from https://www.thestar.co.uk/news/crime/chinese-student-attacked-sheffield-over-coronavirus-1381837

[gwao12523-bib-0022] Lee, K. (2013). Why Asian female stereotypes matter to all: Beyond black and white, east and west. Critical Philosophy of Race, 1(1), 86–103.

[gwao12523-bib-0023] Lee, M. (2020). Corona virus fears show how ‘model minority’ Asian American become the ‘yellow peril’. *NBC News.* Retrieved from https://www.nbcnews.com/think/opinion/coronavirus-fears-show-how-model-minority-asian-americans-become-yellow-ncna1151671

[gwao12523-bib-0024] Liu, H. (2017a). Undoing whiteness: The Dao of anti‐racist diversity practice. Gender, Work and Organization, 24(5), 457–471. 10.1111/gwao.12142

[gwao12523-bib-0025] Liu, H. (2017b). Beneath the white gaze: Strategic self‐orientalism among Chinese Australians. Human Relations, 70(7), 781–804. 10.1177/0018726716676323

[gwao12523-bib-0026] Lorde, A. (2017). Women in culture: An intersectional anthology for gender and women's studies (2nd ed.). Chichester, UK: Wiley Blackwell.

[gwao12523-bib-0027] Mukkamala, S. , & Suyemoto, K. L. (2018). Racialized sexism/sexualized racism: A multimethod study of intersectional experiences of discrimination for Asian American women. Asian American Journal of Psychology, 9(1), 32–46. 10.1037/aap0000104

[gwao12523-bib-0028] Office for National Statistics . (2019). Ethnicity pay gaps in Great Britain. Retrieved from https://www.ons.gov.uk/employmentandlabourmarket/peopleinwork/earningsandworkinghours/articles/ethnicitypaygapsingreatbritain/2018

[gwao12523-bib-0029] Sealey‐Huggins, L. (2018). The climate crisis is a racist crisis: Structural racism, inequality and climate change. In A. Johnson , R. Joseph‐Salisbury , & B. Kamunge (Eds.), The fire now: Anti‐racist scholarship in times of explicit racial violence (pp. 99–113). London, UK: Zed Books.

[gwao12523-bib-0030] Shirazi, Q. (2018). Ambivalent identity and liminal spaces: Reconfiguration of national and diasporic identity in Mohsin Hamid's *The Reluctant Fundamentalist* . South Asian Diaspora, 10(1), 15–29. 10.1080/19438192.2017.1396013

[gwao12523-bib-0031] Yam, K. (2020). Bruce Lee's daughter on ‘kung flu’: ‘My father fought against racism in his movies. Literally’. *NBC News.* Retrieved from https://www.nbcnews.com/news/asian-america/bruce-lee-s-daughter-kung-flu-my-father-fought-against-n1232346

[gwao12523-bib-0032] Yeh, D. (2014). Contesting the ‘model minority’: Racialization, youth culture and ‘British Chinese’/‘Oriental’ nights. Ethnic and Racial Studies, 37(7), 1197–1210. 10.1080/01419870.2014.859288

[gwao12523-bib-0033] Young, S. L. (2015). It's not just black and white. Departures in Critical Qualitative Research, 4(4), 8–32. 10.1525/dcqr.2015.4.4.8

